# Chromatin-Spliceosome Mutations in Acute Myeloid Leukemia

**DOI:** 10.3390/cancers13061232

**Published:** 2021-03-11

**Authors:** Yotaro Ochi, Seishi Ogawa

**Affiliations:** 1 Department of Pathology and Tumor Biology, Graduate School of Medicine, Kyoto University, Kyoto 606-8501, Japan; ochi.yotaro.24v@kyoto-u.jp; 2 Department of Hematology and Oncology, Graduate School of Medicine, Kyoto University, Kyoto 606-8507, Japan; 3 Institute for the Advanced Study of Human Biology (WPI-ASHBi), Kyoto University, Kyoto 606-8501, Japan; 4 Department of Medicine, Centre for Hematology and Regenerative Medicine, Karolinska Institute, Stockholm 171 77, Sweden

**Keywords:** acute myeloid leukemia, myelodysplastic syndrome, splicing factor, cohesin, chromatin, epigenetic regulation, hematopoiesis

## Abstract

**Simple Summary:**

Recent genomic studies have identified chromatin-spliceosome (CS)-acute myeloid leukemia (AML) as a new subgroup of AML. CS-AML is defined by several mutations that perturb epigenetic regulation, such as those affecting splicing factors, cohesin components, transcription factors, and chromatin modifiers, which are also frequently mutated in other myeloid malignancies, such as myelodysplastic syndrome and secondary AML. Thus, these mutations identify myeloid neoplasms that lie on the boundaries of conventional differential diagnosis. CS-AML shares several clinical characteristics with secondary AML. Therefore, the presence of CS-mutations may help to better classify and manage patients with AML and related disorders. The aim of this review is to discuss the genetic and clinical characteristics of CS-AML and roles of driver mutations defining this unique genomic subgroup of AML.

**Abstract:**

Recent genetic studies on large patient cohorts with acute myeloid leukemia (AML) have cataloged a comprehensive list of driver mutations, resulting in the classification of AML into distinct genomic subgroups. Among these subgroups, chromatin-spliceosome (CS)-AML is characterized by mutations in the spliceosome, cohesin complex, transcription factors, and chromatin modifiers. Class-defining mutations of CS-AML are also frequently identified in myelodysplastic syndrome (MDS) and secondary AML, indicating the molecular similarity among these diseases. CS-AML is associated with myelodysplasia-related changes in hematopoietic cells and poor prognosis, and, thus, can be treated using novel therapeutic strategies and allogeneic stem cell transplantation. Functional studies of CS-mutations in mice have revealed that CS-mutations typically cause MDS-like phenotypes by altering the epigenetic regulation of target genes. Moreover, multiple CS-mutations often synergistically induce more severe phenotypes, such as the development of lethal MDS/AML, suggesting that the accumulation of many CS-mutations plays a crucial role in the progression of MDS/AML. Indeed, the presence of multiple CS-mutations is a stronger indicator of CS-AML than a single mutation. This review summarizes the current understanding of the genetic and clinical features of CS-AML and the functional roles of driver mutations characterizing this unique category of AML.

## 1. Introduction

Acute myeloid leukemia (AML) is a group of myeloid malignancies characterized by expanded, undifferentiated myeloid precursor cells and impaired hematopoiesis, however with highly variable clinical manifestations. Despite considerable improvement in the response to initial remission induction chemotherapy, many patients develop refractory disease or experience relapse even after achieving complete remission (CR), which underlines the unmet need for better management and novel therapeutic strategies [1,2]. Although AML was initially classified based on morphological features of leukemic cells, the discovery of recurrent cytogenetic abnormalities and driver mutations has substantially promoted our understanding of AML pathogenesis. Particularly, driver mutations are now known to play a central role in the evolution of AML and have been incorporated into the definition of different AML subtypes according to the World Health Organization (WHO) classification [3,4] of myeloid neoplasms and acute leukemia.

Recent genetic studies using next-generation sequencing (NGS) technologies have almost completely cataloged driver mutations in AML [4,5]. Through the analysis of these mutations in a large cohort of patients, a novel genomic classification of AML was proposed, which comprises 11 unique subgroups with distinct molecular and prognostic features [6]. Among these subgroups, a novel category was defined as “AML with mutations in genes encoding chromatin and/or RNA splicing regulators” (hereafter called chromatin-spliceosome (CS)-AML), which is yet to be fully characterized. Mutations defining CS-AML (hereafter referred to as CS-mutations) consist of *RUNX1*, *ASXL1*, *BCOR*, *STAG2*, *EZH2*, *SRSF2*, *SF3B1*, *U2AF1*, *ZRSR2*, and partial tandem duplication (PTD) of *KMT2A* (*MLL*) gene (*MLL*-PTD) [6]. Importantly, these mutations affecting epigenetic regulators are also frequently found in myelodysplastic syndrome (MDS) and secondary AML (sAML) (or AML with myelodysplasia-related changes), a subtype of AML that develops after antecedent hematological diseases (Table 1) [7,8,9,10,11,12], suggesting that these mutations define a group of mutually overlapping myeloid neoplasms with unique pathophysiology distinct from typical de novo AML. Clinically, CS-AML correlates with progressive myelodysplasia-related changes in hematopoietic cells and poor prognosis [6].

In terms of the functional implications of CS-mutations, recent studies have demonstrated that these mutations cause abnormal hematopoiesis and leukemogenesis by altering the epigenetic regulation of target genes [13,14,15,16,17,18,19,20,21]. Typically, in vivo mouse models with single CS-mutation show relatively mild abnormalities in hematopoiesis. Meanwhile, multiple CS-mutations often synergistically cause more prominent phenotypes in mice, such as the development of lethal MDS/AML, underscoring the significance of accumulation of multiple CS-mutations in the pathogenesis of MDS/AML [22,23,24].

In this review, we summarise and discuss the current understanding of the genetic and clinical characteristics of CS-AML and the functional roles of the relevant mutations in the pathogenesis of this unique category of AML.

## 2. Genetics of Chromatin-Spliceosome Acute Myeloid Leukemia (CS-AML)

### 2.1. Genetic Classification of AML

The molecular pathogenesis of AML, initially studied by cytogenetic analysis, is currently assessed by examining recurrent chromosomal abnormalities, such as t(15;17) and t(8;21), which are efficient diagnostic and prognostic markers for AML [1]. However, nearly half of all AML patients show a normal karyotype without apparent chromosomal structural abnormalities [25]. Given this background, The Cancer Genome Atlas (TCGA), a landmark cancer genomics program, analyzed the genomes of 200 patients with AML using whole-genome sequencing (WGS) (50 cases) and whole-exome sequencing (WES) (150 cases), together with RNA, microRNA, and DNA methylation analysis, to comprehensively identify genetic and epigenetic abnormalities in AML [5]. The number of coding mutations, including single nucleotide variations and insertions/deletions, was 13 per patient, on average. This project also identified 23 significantly mutated genes, including previously known driver genes, such as *DNMT3A*, *FLT3*, *NPM1*, *IDH1*, *IDH2*, and *CEBPA*, as well as genes recently implicated in AML leukemogenesis, such as *U2AF1*, *SRSF2*, *EZH2*, *SMC1A*, and *STAG2*. Almost all samples had at least one nonsynonymous mutation in one of nine functional categories of genes relevant to AML pathogenesis, including transcription factor fusions (e.g., t(15;17), t(8;21), and inv(16)/t(16;16)) (18%), signaling genes (e.g., *FLT3*, *KIT*) (59%), DNA methylation-related genes (e.g., *DNMT3A* and *TET2*) (44%), chromatin-modifying genes (e.g., *ASXL1* and *EZH2*) (30%), the gene encoding nucleophosmin (*NPM1*) (27%), myeloid transcription factor genes (e.g., *RUNX1* and *CEBPA*) (22%), tumor suppressor genes (e.g., *TP53* and *WT1*) (16%), spliceosome genes (e.g., *SRSF2* and *U2AF1*) (14%), and cohesin complex genes (e.g., *STAG2* and *SMC1A*) (13%). Thus, driver events have been identified in most AML cases using integrated NGS and cytogenetic analysis in recent years.

A landmark study analyzed a large cohort of 1540 patients with AML using targeted-capture sequencing of 111 driver genes to understand how genetic diversity defines AML pathogenesis and informs clinical practice [6]. This identified over 5000 driver mutations across 76 genes or genomic regions, with mutations in two or more drivers discovered in 86% of patients. Intriguingly, the patterns of co-occurrence and mutual exclusivity of genetic alterations stratified the cohort into 11 categories with distinct diagnostic features and prognosis. In addition to previously defined AML subgroups, such as AML with mutated *NPM1* or biallelic mutated *CEBPA*, three new heterogeneous genomic categories emerged in this study. These included CS-AML (18%), AML with *TP53* mutations and/or chromosomal aneuploidies (13%), and, AML with *IDH2*^R172^ mutations (without other class-defining abnormalities; 1%). This classification strategy uniquely defined approximately 80% of AML into a single group, depending solely on the presence of genetic abnormalities. CS-AML was associated with old age and poor clinical outcome, with 20% of CS-AML patients having a pre-existing myeloid disorder or dysplasia. Due to limited available data on this new category, CS-AML cases have not been assigned a specific risk group by the European Leukemia Net (ELN) guideline, which defines risk stratification by genetics [2].

Another recent study analyzed 672 samples collected from a cohort of 562 AML patients (Beat AML program) using WES, RNA-sequencing, and assaying ex vivo drug sensitivity for 122 small-molecule inhibitors [26]. The Beat AML and TCGA studies, both of which analyzed AML mutations by WES or WGS, generally showed similar frequencies of driver mutations. Interestingly, the strength of drug responses correlated with mutational status, thus implying that the classification of AML patients based on mutational profiles may prove useful for risk prediction and application of precision medicine.

In summary, recent large genomic studies have helped catalog a list of driver mutations in AML, most of which occur with the acquisition of at least one genetic abnormality. The molecular findings of these studies inform disease classification, risk prediction, and facilitate better clinical management, including the application of novel molecular therapies.

### 2.2. Definition of CS-AML

**Figure 1:**
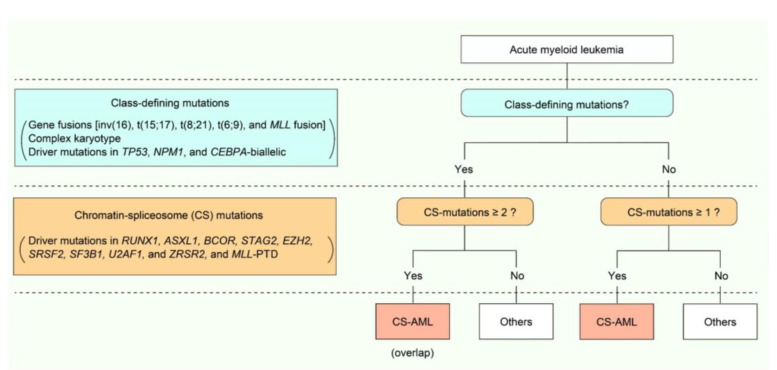
Definition and genetic features of chromatin-spliceosome acute myeloid leukemia (CS-AML). Scheme demonstrating the definition of CS-AML.

CS-AML is defined by one or more mutations in genes encoding proteins involved in chromatin regulation or the splicing machinery pathway. These CS-mutations include those in *RUNX1*, *ASXL1*, *BCOR*, *STAG2*, *EZH2*, *SRSF2*, *SF3B1*, *U2AF1*, *ZRSR2*, and *MLL*-PTD [6]. In combination with other class-defining mutations, such as t(15;17), t(8;21), inv(16), t(6;9), *KMT2A* fusion genes, complex karyotype, or driver mutations in *NPM1*, *TP53*, or *CEBPA* biallelic mutations, two or more of the CS-mutations are required to define CS-AML (Figure 1). Thus, CS-AML is a subtype of AML defined solely by genetic profiles, and not by a single mutation. In the following sections, we describe the functional roles of driver mutations in CS-AML pathogenesis as well as associated genetic and clinical features in detail.

## 3. Driver Genes Mutated in CS-AML

### 3.1. Splicing Factors (SF)

**Figure 2:**
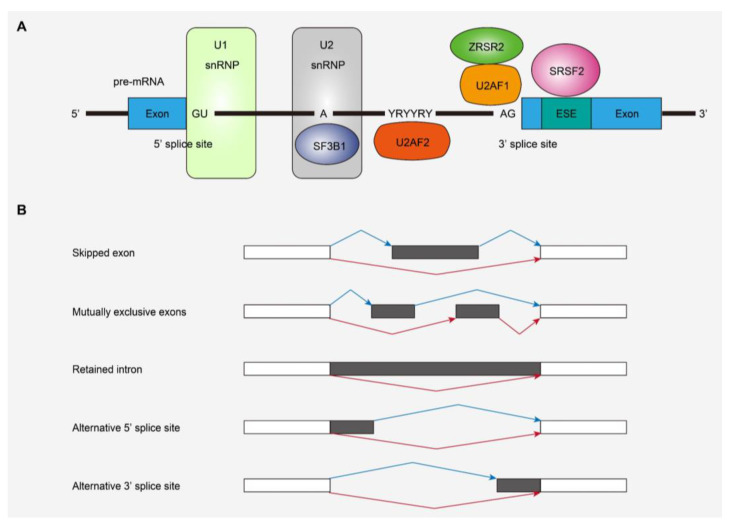
Splicing factors (SFs) and alternative splicing affected by mutations. (**A**) SFs frequently affected in myeloid malignancies and their functions in RNA splicing. (**B**) Types of alternative splicing events detected in SF-mutated leukemias. snRNP, small nuclear ribonucleoprotein; ESE, exonic splicing enhancer.

SF mutations were first reported in MDS and other myeloid neoplasms, as well as chronic lymphocytic leukemia, and represent a novel class of driver mutations in cancers [27,28]. As described above, SF mutations are one of the major driver mutations in CS-AML [6,7,8,9,10], and have been found in various myeloid neoplasms, such as MDS, AML, and myeloproliferative neoplasms (MPNs) [7,8,9,10,11]. Frequently affected SF genes include *SF3B1*, *SRSF2*, *U2AF1*, and *ZRSR2*, all of which are included in class-defining mutations for CS-AML (Figure 2A) [29]. In the former three genes, mutations change specific amino acid residues, suggesting the gain-of-function nature of those mutations [27]. In contrast, most mutations in *ZRSR2*, located on the X chromosome, are nonsense or frameshift mutations, suggesting inactivation of the gene function [27]. SF mutations are almost always heterozygous (or hemizygous) and seen in a mutually exclusive manner, suggesting that multiple abnormalities in the vital splicing pathway are not compatible with the survival and clonal selection of leukemia cells [27].

Previous studies have revealed that SF mutations generally induce widespread RNA splicing alterations, the patterns of which depend on the mutated SFs (Figure 2B). Specific splicing alterations induced by each SF mutation have been vigorously studied using in vitro and in vivo models of SF-mutated leukemia as well as transcriptome studies of human leukemia samples with SF mutations. Mutant *SF3B1* induces the usage of alternative branch points and causes an alternative 3’-splice site [30,31]. *SRSF2* and *U2AF1* mutations have been shown to cause alternative exon usage [13,32,33,34]. The *ZRSR2* mutation involves retention of minor (U12-type) introns that make up less than 1% of introns in humans, while splicing of major (U2-type) introns is not significantly affected [35]. These specific patterns of alternative splicing due to SF mutations are also observed in primary human leukemia samples [13,14,15,36]. SF mutations lead to mis-splicing of key hematopoietic regulators, such as *EZH2* and *INTS3* in *SRSF2*-mutated MDS and *ERFE, BRD9,* and *SF3B1* in *SF3B1*-mutated MDS, which may partially explain the disease phenotypes caused by SF mutations [13,37,38,39,40]. Thus, SF mutations included in CS-mutations exert differential effects on global RNA splicing, indicating that not all SF mutations cause leukemogenesis in the same way. In this regard, there may be an overlapping mechanism involved in the development of leukemia caused by SF mutations. An intriguing report demonstrated that SF mutations in *SRSF2* and *U2AF1* induce cell growth defects through elevated levels of R-loops, replication stress, and ATR-Chk1 activation [41]. As enhanced R-loops correlated with compromised proliferation of blood progenitors derived from the bone marrow, which was rescued by forced RNase H expression, they may contribute to aberrant hematopoiesis and leukemogenesis [41].

In mouse models, *SF3B1*, *SRSF2*, and *U2AF1* mutations cause not only splicing changes but also aberrant hematopoiesis and/or MDS-like phenotypes, confirming the functional importance of these mutations in hematopoiesis and leukemogenesis [13,14,15]. A recent report also demonstrated that *SRSF2* and the isocitrate dehydrogenase gene, *IDH2*, mutations frequently co-exist in human AML cases, and that co-expression of mutant SRSF2 and IDH2 caused lethal MDS/MPN-like diseases with myelodysplasia and proliferative features in mice, possibly through the synergistic effects of these mutations on the epigenome and RNA splicing [39]. Thus, SFs contribute to leukemogenesis by affecting epigenetic regulation and alternative splicing.

### 3.2. Cohesin Complex

**Figure 3:**
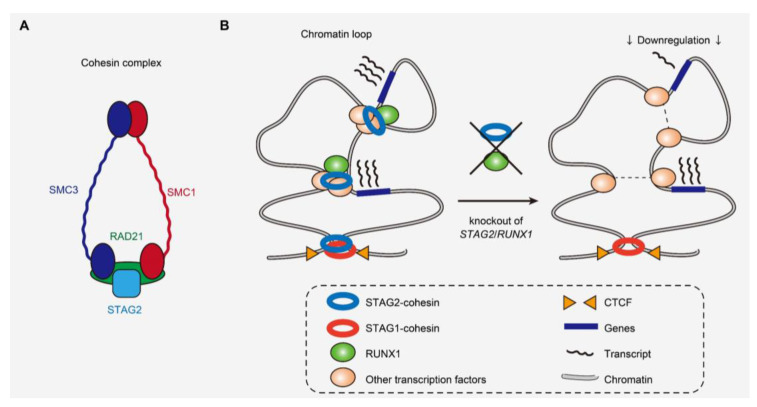
Cohesin complex and the 3D genome structure affected by cohesin and *RUNX1* mutations. (**A**) Cohesin complex and its components frequently mutated in myeloid malignancies. (**B**) Scheme demonstrating the perturbation of short-range loops and deregulation of a subset of genes caused by loss of STAG2 and RUNX1 [22].

Cohesin mutations also represent a novel class of driver mutations in cancers (Figure 3A) [5,42]. Comprised of STAG1 or STAG2, RAD21, SMC1, and SMC3, the cohesin complex is involved in multiple different cellular functions, such as maintaining sister chromatid cohesion during cell division, and DNA repair [43]. Moreover, cohesin has recently also been implicated in the maintenance of the 3D genome architecture to regulate gene transcription [44]. Cohesin mutations are found in ~10–15% of AML, MDS, and MPN cases [42,45,46]. Interestingly, cohesin mutations are particularly frequent in Down syndrome-related acute megakaryoblastic leukemia, in which together with *CTCF* mutations, cohesin mutations were found in >50% of the cases, although rarely found in transient myeloid disease [47]. A member of the cohesin complex, *STAG2* is also mutated in bladder cancers [48,49,50]. Mutations have been reported to affect all cohesin components, such as *STAG2*, *RAD21*, *SMC1*, and *SMC3* in a mutually exclusive manner [51]. Among cohesin mutations, *STAG2* mutation is most frequent and is one of the class-defining mutations in CS-AML [6,42]. Most *STAG2* mutations are nonsense or frameshift, predicted to cause protein truncation and loss-of-function [42].

Several studies have illustrated that cohesin mutations cause enhanced self-renewal and aberrant differentiation of hematopoietic stem and progenitor cells (HSPCs) in both in vitro and in vivo models [16,17,22,52,53,54]. Cohesin mutations particularly alter transcriptional regulation of several transcription factors, such as RUNX1, GATA2, and ERG in hematopoietic cells [16,17,22,52,53]. Moreover, recent research showed that STAG2 loss in mice preferentially disrupted short-range 3D chromatin interactions [22,53]. Genes showing high basal levels of transcriptional pausing or those regulated by super-enhancers were particularly prone to downregulation, indicating that disruption of chromosomal interactions does not necessarily result in global downregulation of gene expression (Figure 3B) [22]. The genes downregulated by cohesin deficiency included those involved in the interferon response pathway, downregulation of which is also observed in human leukemias with cohesin mutations [22,55]. In the background of STAG2 absence, additional STAG1 loss abrogated hematopoiesis, consistent with the reported synthetic lethality between STAG1 and STAG2 in leukemia cell lines and the redundancy in STAG1 and STAG2 function in chromatid segregation [53,56,57].

*STAG2* mutations are almost always accompanied by other driver mutations, such as in *RUNX1*, *SRSF2*, and *ASXL1* [6,8,9,22], but how cohesin mutations induce myeloid neoplasms in conjunction with other driver mutations remains to be determined. A recent study showed that combined loss of STAG2 and RUNX1, that colocalize at enhancer regions, synergistically attenuated enhancer–promoter loops and caused lethal MDS-like phenotypes in mice (Figure 3B) [22]. Another report demonstrated the interaction of cohesin with the chromatin modifying protein ASXL1, which also supports the functional relationship of cohesin with other driver genes frequently co-mutated with cohesin [58]. These studies underscore the importance of understanding cohesin function in the context of co-mutations in human leukemias to further elucidate the functional relationship between different driver genes that could synergistically affect common molecular processes.

### 3.3. Transcription Factors

Transcription factors regulate gene expression by binding to specific DNA sequences, and are frequent targets of genetic alterations in cancers, including AML. Representative mutational targets include *RUNX1*, *CEBPA*, and *GATA2*, of which *RUNX1* is included in genes defining CS-AML. *RUNX1* mutations have frequently been identified in a variety of hematological malignancies, including AML, MDS, MPN, and acute lymphoblastic leukemia (ALL) [4,5,6,7,8,9,10,59,60,61,62,63]. *RUNX1* mutations are also found in the germline and cause familial platelet disorder with a predisposition to AML [64,65]. AML accompanied with mutated *RUNX1* is assigned to the adverse prognosis group based on the ELN guidelines [2]. *RUNX1* is also affected by more than 50 chromosomal translocations [66], such as t(8;21) and t(3;21), that generate *RUNX1*-*RUNX1T1* and *RUNX1*-*MECOM*, respectively and are commonly involved in AML [66,67,68].

RUNX1 is a master transcription factor of hematopoietic cells that plays crucial roles in embryogenesis, and both definitive and adult hematopoiesis in vertebrates [18,19,20,69,70,71,72,73]. Functional studies have shown that RUNX1 regulates the transcription of key genes in hematopoiesis, such as *KIT*, together with other hematopoietic transcription factors, such as GATA proteins and SPI1, and mutated RUNX1 or RUNX1 fusion proteins deregulate target genes to cause aberrant hematopoiesis [59,74,75,76]. In several mouse models, the loss of RUNX1 alone does not seem to cause phenotypes of hematological malignancies [18,19,20]. However, in bone marrow transplantation models, the RUNX1 mutant causes MDS in collaboration with EVI1 [77,78]. Moreover, recent studies have shown the interaction of *RUNX1* with other frequently co-mutated drivers, such as *STAG2* and *ASXL1*, thus promoting the advancement of myeloid neoplasms [22,23]. In the Beat AML study describing drug sensitivity assays performed ex vivo on primary leukemia cells, *RUNX1*-mutated AML showed higher sensitivity to PIK3C and mTOR inhibitors and to the multi-kinase vascular endothelial growth factor receptor (VEGFR) inhibitor, suggesting the possibility of targeted therapy for mutated *RUNX1*; however, this remains to be validated in vivo [26].

### 3.4. Chromatin Modifiers

Genes related to the Polycomb group (PcG) of proteins are frequently affected in myeloid neoplasms. In mammals, there are two major PcG complexes, namely polycomb repressive complex 1 (PRC1) and 2 (PRC2), which regulate ubiquitination and methylation of histone modifications, respectively [79,80,81,82]. *EZH2*, which encodes a component of PRC2 complex, is mutated in hematological neoplasms, such as AML, MDS, MPN, and B-cell lymphoma [5,6,8,10,83,84,85]. Loss-of-function mutations of *EZH2* reduce global H3K27me3 levels to deregulate expression of target genes, and result in the development of MDS, MDS/MPN, and T-cell acute lymphoblastic leukemia [24,86]. In a mouse model with a *JAK2* mutation that developed MPN, additional loss of PRC2 increased sensitivity to bromodomain inhibition [87]. In the background of EZH2 deficiency, EZH1 becomes essential for maintaining hematopoiesis, indicating the synthetic lethality between EZH1 and EZH2 [86,88].

BCOR and BCORL1 proteins function as components of PRC1.1, a noncanonical PRC1, and are frequent targets of somatic mutations in AML, sAML, MDS, and chronic myelomonocytic leukemia (CMML) [6,7,8,9,10,89,90]. BCOR loss in mice promotes myeloid cell proliferation and differentiation along with an upregulation of HoxA genes [91,92]. Combined loss of BCOR and TET2 causes lethal MDS in mice [92]. The Beat AML program, a functional genomics study of AML, suggests that patients with *BCOR* mutations show higher sensitivity to JAK inhibitors compared to those with *BCOR* wild-type, which remains to be confirmed in future studies [26]. Interestingly, patients carrying both *BCOR* and *RUNX1* mutations, both of which are members of CS-mutations, were particularly sensitive to JAK inhibitors, indicating that cooperation between CS-AML drivers may influence therapeutic strategies.

ASXL1, an epigenetic modulator, interacts with EZH2 [93]. Mutations in *ASXL1* are frequently found in a variety of myeloid neoplasms, such as MDS, AML, MPNs, and CMML, as well as age-related clonal hematopoiesis in healthy individuals [5,6,8,9,10,94,95,96,97,98]. AML with mutated *ASXL1* is assigned to the adverse prognosis group by the ELN genetic risk stratification guidelines [2]. Studies investigating *ASXL1* function have suggested that *ASXL1* mutations alter the pattern of histone modifications, such as H3K4me3, H3K27me3, and H2AK119Ub, and impair hematopoietic function [93,96,99,100,101]. Perturbed expression of ASXL1 induces MDS- or MPN-like phenotypes in several in vivo models [21,102,103,104]. Moreover, mutations in *ASXL1* and *SETBP1* induce leukemic transformation and MDS in a mouse model [105]. The combination of *SETBP1* and *ASXL1* mutations is seen in patients with germline *GATA2* mutation-related MDS, in whom monosomy 7 occurred as an early somatic event followed by the acquisition of both mutations [106]. Thus, combined mutations of *SETBP1* and *ASXL1* seem to synergistically induce myelodysplasia. Molecules with therapeutic properties, including BRD4 and HDAC inhibitors, that reverse the action of mutant *ASXL1* have been investigated, which suggests the potential treatment strategy against *ASXL1*-mutated leukemia [26,103,107].

*MLL*-PTD abnormality is found in a subset of MDS and AML, which typically shows normal cytogenetics or trisomy 11 [6,108,109,110,111,112,113,114]. Particularly, patients with acute erythroid leukemia, a subtype of AML characterized by proliferation of erythroid and myeloid blast cells in the bone marrow, carry this abnormality more frequently compared to other non-erythroid AML [115]. Functional studies have shown that *MLL*-PTD mutation results in aberrant chromatin remodeling and alters the transcription of target genes, and causes expansion of HSPCs and enhanced colony-formation [109,116,117,118,119]. However, *MLL*-PTD abnormality alone is not sufficient to develop leukemia in mice [116,117], suggesting that additional mutations together with *MLL*-PTD are necessary for leukemic transformation. In this regard, considerable co-occurrence of *MLL*-PTD and *STAG2* mutations in acute erythroid leukemia [115], both of which are CS-mutations, are interesting avenues to be explored in future functional studies.

## 4. Clinical Features of CS-AML

**Figure 4:**
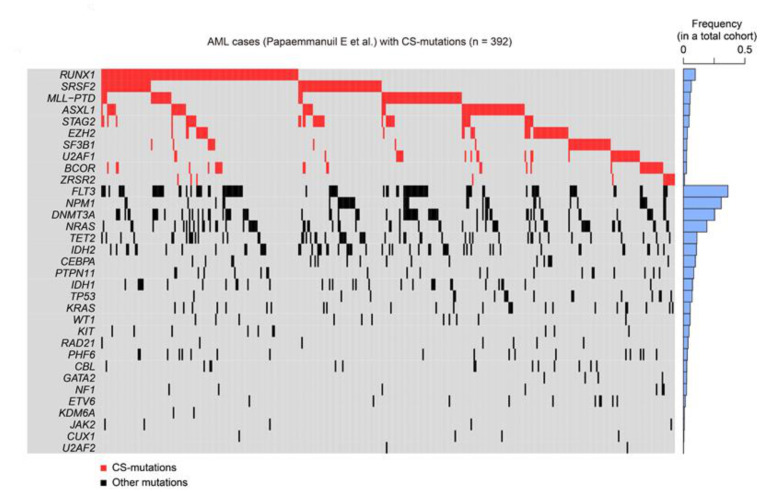
CS-mutations in AML. Heatmap representing CS-mutations found in a previous study [6]. Blue bars on the right show the frequency of each mutation in a large cohort of patients with AML (1540 cases). Not all cases demonstrated here satisfy the definition of CS-AML, as a subset of AML with CS-mutations is excluded from CS-AML due to presence of other class-defining mutations, as shown in Figure 1.

CS-AML is defined by mutations in several genes implicated in epigenetic regulation. To fully understand the molecular features of CS-AML, it is worth noting that CS-mutations are also frequently found in other myeloid tumors, such as sAML and MDS (Table 1) [7,8,9,10,11]. Another interesting feature of CS-mutations is the frequent co-occurrence of these mutations. For instance, all cases with mutated *STAG2* had another CS-mutation in the original report defining CS-AML [6], suggesting that co-existence of multiple driver mutations plays a crucial role in the development of this type of leukemia (Figure 4). 

A previous study analyzed mutations in 194 patients with sAML or therapy-related AML and 105 de novo AML cases [7]. This study demonstrated that the presence of one or more mutations in *SRSF2*, *SF3B1*, *U2AF1*, *ZRSR2*, *ASXL1*, *EZH2*, *BCOR*, or *STAG2*, called “secondary-type” mutations, was highly specific to sAML. Notably, all secondary-type mutations are included in CS-mutations. These secondary-type mutations define a distinct genetic subgroup that has poor prognosis in elderly patients with de novo AML, suggesting that secondary-type mutations identify sAML-like disease within de novo AML [7]. Another study investigating gene expression and DNA methylation profiles in leukemia revealed that SF-mutant MDS and SF-mutant AML were clinically, cytologically, and molecularly similar [12]. This suggests that SF-mutant MDS/AML may be considered as myeloid disorders lying on the boundaries of MDS and AML. Collectively, CS-AML shares similar molecular features with MDS and sAML.

According to the report that defined CS-AML, CS-AML accounts for approximately one-fifth (18%) of AML, the second largest among the 11 subgroups [6]. This was corroborated by a recent study from the Northern Italy Leukemia Group (NILG), which analyzed a prospective cohort of 413 patients with de novo AML enrolled in a randomized trial and reported a similar frequency of CS-AML in those patients (17.6%) [120]. This study also revealed that CS-AML shared clinical characteristics with sAML (older age, lower white blood cell counts, and higher rate of multilineage dysplasia), and was associated with adverse prognosis compared to other de novo AML (overall survival, 30% in CS-AML and 17% in sAML vs. 61% in other de novo AML). Importantly, allogeneic stem cell transplantation after the first CR improved survival in both de novo AML within CS-AML category and sAML. This study emphasizes the clinical significance of diagnosing CS-AML for improving prognosis by application of therapeutic strategies such as allogeneic stem cell transplantation.

**Figure 5:**
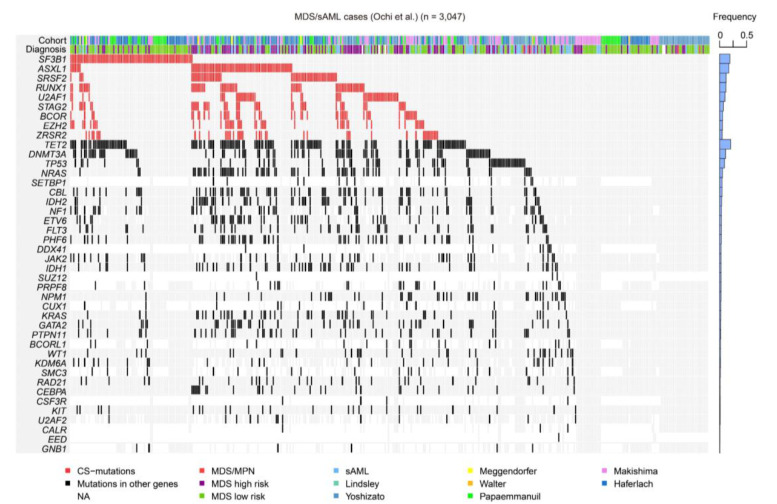
CS-mutations in MDS/sAML. A heatmap representing the mutations found in a large cohort of MDS, related myeloid disorders, and sAML, as shown in our previous study [22]. Blue bars on the right show frequencies of mutations in all cases. MPN, myeloproliferative neoplasm; NA, not available.

Despite being among the major driver mutations in both MDS and de novo AML, CS-mutations are more frequently seen in the former than in the latter (Table 1) [8,9,10]. In a meta-analysis of 3047 patients with MDS, related myeloid disorders, and sAML, approximately half of them carried at least one CS-mutation, suggesting that these mutations play a central role in MDS and AML pathogenesis (Figure 5) [22]. Furthermore, this study demonstrated that CS-mutations in four representative genes (*STAG2*, *RUNX1*, *SRSF2*, and *ASXL1*, called “*SRSA*” genes) were conspicuously enriched and co-occurred in both MDS and sAML [22]. At least one of these genes was found mutated in 31.8% of patients, and 46.8% of those carried mutations in two or more *SRSA* genes. More *SRSA* mutations were found in MDS patients that, importantly, increased the likelihood of transformation to sAML in a linear manner; 5.1%, 11.1%, and 32.9% of patients with MDS carrying 0, 1, or ≥2 *SRSA* mutations, respectively, experienced transformation to sAML. Consistently, patients with more than one *SRSA* mutation had a poorer overall survival than those with only one, which also negatively affected survival. The variant allele frequencies of the *SRSA* mutations suggested that *SRSF2* mutations were acquired earlier than other mutations, followed by *RUNX1*, *STAG2*, and *ASXL1* mutations [22]. The co-existence of *SRSA* mutations was also confirmed within CS-AML cases in an AML cohort at a similar frequency as that reported above [6,22]. This study revealed similar patterns of co-occurrence of particular mutations in a subset of MDS and AML, and identified those that induced leukemic transformation of MDS. Importantly, the presence of two or more CS-mutations is a stronger indicator to define CS-AML than a single mutation when other category-defining genetic events exist, such as transcription factor fusions (Figure 1) [6].

In summary, CS-AML is a genomic subgroup of AML defined by several gene mutations, which are also frequently identified in MDS and sAML. These mutations may identify myeloid neoplasms lying on the boundaries of conventional categorization of AML. CS-AML exhibits clinical characteristics similar to those of sAML, and the unambiguous presence of CS-mutations may help clinicians to better classify and manage patients with AML, in the absence of detailed clinical history, and apparent morphological or cytogenetic abnormalities. The accumulation of multiple CS-mutations also seems to be important for the development and progression of AML, as multiple CS-mutations, such as in *STAG2*, *RUNX1*, *SRSF2*, and *ASXL1*, preferentially co-occur in patients with high-risk MDS who later develop sAML.

## 5. Conclusions and Future Perspectives

CS-AML is a novel form of AML and is characterized by mutations in several genes, such as those encoding the spliceosome, cohesin complex, transcription factors, and chromatin modifiers, all of which are implicated in various epigenetic pathways. Class-defining mutations in CS-AML are also frequently found in MDS and sAML, indicating a molecular similarity among a subset of CS-AML, MDS, and sAML. Clinically, CS-AML correlates with myelodysplasia-related changes in hematopoietic cells and poor patient prognosis, and has been successfully treated by allogeneic stem cell transplantation, with the potential of using novel therapeutics. Notably, the mutational status in patients with AML is associated with sensitivity to various anticancer drugs, which suggests the need to adjust treatment based on the mutational status [26].

Although the functional role of each CS-mutation in hematopoiesis and leukemogenesis is not completely homogenous, at least some aspects are shared between mutations. Functional studies using mouse models have revealed that CS-mutations typically perturb epigenetic regulation and transcription, and impair hematopoietic functions, causing aberrant hematopoiesis and/or MDS-like phenotypes. Moreover, multiple CS-mutations in mice often synergize to induce more severe phenotypes in the hematopoietic system, such as the development of lethal MDS/AML, suggesting that the accumulation of abnormalities in genes involved in epigenetic pathways may play a critical role in the development and progression of a subset of MDS/AML.

Several questions remain to be addressed to better understand CS-AML. For instance, the distinct diagnostic boundaries between CS-AML, MDS, and sAML become obscure when considering the similarity in the molecular signatures of these diseases. This ambiguity hinders the development of optimal diagnosis and therapeutic strategies for patients carrying CS-mutations. Future studies on pan-myeloid tumors should identify ways to integrate the conventional morphological and clinical diagnosis with the mutational profiles to optimize patient diagnosis and disease management. Furthermore, the molecular mechanism by which distinct CS-mutations induce comparable phenotypes and define the same disorder remains nebulous. As CS-mutations may alter epigenetic regulation, integrative analysis of genomic and epigenomic alterations in AML may prove the common molecular pathogenesis of CS-AML. Recent work suggests applying precision medicine that targets CS-mutations, which, although promising and potentially effective, warrants validation in multiple systems before translating to human trails. Given that CS-AML and MDS share common molecular signatures, patients with CS-AML may be treated using the hypomethylating agents azacitidine and decitabine that have been used in MDS treatment. Future studies should comprehensively identify the landscape of genetic and epigenetic features that lead to the pathogenesis of CS-AML, which can be used to develop novel therapeutic strategies for this unique category of AML.

## Figures and Tables

**Table 1:** Mutations defining chromatin-spliceosome acute myeloid leukemia (CS-AML).

Driver Mutations	Pathway/Functions	Approximate Frequency (%)		
		de novo AML	sAML	MDS
*SRSF2*	Spliceosome	2–7	12–20 *	12–17 †
*SF3B1*	Spliceosome	2–10	7–11 *	13–33 †
*U2AF1*	Spliceosome	1–4	11–16 *	5–11 †
*ZRSR2*	Spliceosome	0–1	3–8 *	3–8 †
*STAG2*	Cohesin	2–7	10–14 *	3–8 †
*RUNX1*	Transcription factor	5–20	20–31 *	6–14
*EZH2*	Chromatin modification	2–4	5–9 *	4–15 †
*BCOR*	Chromatin modification	2–3	7–8 *	2–6 †
*ASXL1*	Chromatin modification	5–15	19–32 *	10–23 †
*MLL*-PTD	Chromatin modification	5–8	14	4–5

* The frequency is significantly higher in secondary acute myeloid leukemia (AML) (sAML) compared with de novo AML, as previously reported [7]. † The frequency is significantly higher in myelodysplastic syndrome (MDS) compared with de novo AML, as previously reported [8].
